# Pedicled medial femoral condyle corticoperiosteal flap for achieving union in patients with nonunion of the distal half of the femur (A short case series of three patients)

**DOI:** 10.1186/s12891-025-08644-6

**Published:** 2025-05-15

**Authors:** Nader Kamiel, Ashraf N Moharram, Ayman Shaheen, Mostafa Ezzat, Walid Ebeid

**Affiliations:** Department of Orthopedic Surgery, Kasr Alainy, Cairo University, Cairo, Egypt

**Keywords:** Recalcitrant nonunion of the distal femur, Pedicled medial femoral condyle flap, Corticoperiosteal graft, Vascularized bone graft

## Abstract

**Background:**

Recalcitrant bone nonunion is characterized by impaired biological potential at the fracture site due to diminished vascularity and loss of osteogenic cells, reducing the success rate of nonvascularized bone grafts. In cases of ununited fractures of the tibia and femur with minimal gapping, the medial femoral condyle (MFC) corticoperiosteal flap offers a promising solution. This study aims to evaluate the effectiveness of the pedicled MFC corticoperiosteal flap in achieving union in recalcitrant nonunion of the distal half of the femur. The secondary objective is to report complications associated with this technique.

**Methods:**

Three male patients with recalcitrant nonunion of the distal half of the femur were included. The transposition ratio was calculated by dividing the distance between the medial femoral epicondyle and the nonunion site (DMEB) by the distance from the medial femoral epicondyle to the apex of the lesser trochanter (DMELT). Patients with a ratio greater than 0.5 were excluded. Each patient underwent adequate rigid fixation, followed by harvesting a pedicled MFC corticoperiosteal flap from the medial distal femur. The flap was rotated to cover the nonunion site and augmented with an iliac crest bone graft to fill residual gaps. Bony union was monitored through monthly X-rays and CT scans.

**Results:**

All three patients (average age 36.7 years) had recalcitrant nonunion, two cases being aseptic atrophic and one septic. Union was achieved in all patients (100% union rate), with an average time to union of 6.7 months. No mechanical failures were observed. Complications included saphenous nerve injury and seroma in one patient, and an incisional hernia at the iliac crest graft donor site in another.

**Conclusion:**

The pedicled MFC-CP flap appears to be a feasible option for treating recalcitrant distal femur nonunion, with minimal donor site morbidity. However, larger studies are needed to confirm its efficacy.

## Introduction

Recalcitrant bone nonunion is defined as the failure of a fracture to unite after at least one nonunion surgery, including nonvascularized iliac crest bone grafting [1, 2]. Surgical options for managing recalcitrant nonunion include the Masquelet technique, vascularized bone flaps, and Ilizarov methods [3–7]. A vascularized fibular flap is an excellent solution for gap nonunions measuring more than 5 cm. However, in cases of recalcitrant nonunion with no gap or small gaps less than 3 cm, it is challenging to identify the entry point of the nutrient artery into the vascularized fibular flap. Consequently, part of the harvested fibula often needs to be discarded to achieve an appropriate fit at the nonunion site, which may compromise the viability of the flap [8–13].

The medial femoral condyle (MFC) corticoperiosteal flap is a suitable option for recalcitrant nonunion with minimal gapping or small gaps of up to 3 cm, particularly when augmented with an iliac crest bone graft. The MFC corticoperiosteal flap is harvested based on the descending genicular vessels, which arise from the femoral artery at the adductor hiatus. These vessels are present in approximately 89% of individuals. In the remaining 11%, the descending genicular vessels are absent, and the superomedial genicular vessels are dominant and supply the medial femoral condyle periosteum [9, 14].

Adequate rigid fixation is paramount in every nonunion case to reduce strain at the fracture gap to less than 10%, facilitating bone formation in accordance with Perren’s strain theory. Atrophic nonunion results from both a lack of biological activity and mechanical stability. Its treatment, therefore, requires the combined use of osteogenic bone flaps and rigid fixation [15–17].

The MFC corticoperiosteal flap can be used in a pedicled fashion for nonunion of the distal half of the femur. It can be harvested easily from the distal aspect of the medial femoral condyle and rotated proximally to cover the nonunion site after fracture fixation [18]. The MFC corticoperiosteal flap provides the fracture site with osteogenic cells and growth factors essential for union. Careful harvesting is critical; the periosteum should be raised with a thin chip of the underlying cortex to avoid damage to the cambium layer.

In this study, we investigated the adequacy of the pedicled MFC corticoperiosteal flap for patients with nonunion of the distal half of the femur. The primary objective was to evaluate the medial femoral condyle corticoperiosteal flap as a viable treatment option for nonunion of the distal half of the femur. The secondary objective was to identify complications related to flap harvesting. We hypothesized that the pedicled MFC corticoperiosteal flap would be a suitable treatment option for patients with recalcitrant nonunion of the distal half of the femur.

## Patients and methods

This prospective short case series included three patients with recalcitrant nonunion of the distal half of the femur. All patients underwent surgical management using a pedicled medial femoral condyle (MFC) corticoperiosteal flap in combination with adequate rigid fixation. Informed consent was obtained from all patients for both the surgical procedure and participation in the study.

### Preoperative data collection

Preoperative data were collected for each patient, including age, sex, site of nonunion, number of previous surgeries, history of infection, prior nonvascularized bone grafting, duration of nonunion, and the size of the nonunion defect. These data are summarized in Table 1.

The study included three male patients, with an average age of 36.7 years (range: 33–43 years). Two patients had nonunion of the right midshaft femur, and one patient had nonunion of the left distal femur metaphysis. The average duration of nonunion was 60 months (range: 30–108 months).

### Detailed case descriptions

In Patient 1, there was a nonunion at the right midshaft femur. Initially, there was a 5 cm bone defect, which was managed with a cement spacer around an interlocking nail. However, progressive weight bearing resulted in proximal migration of the nail due to loss of fixation at the proximal interlocking screw, which was positioned outside the locking hole. This led to shortening and displacement of the cement spacer. At the time of index surgery, the residual defect measured approximately 10 mm at the anterior, medial, and posterior cortices, with a 2 cm defect at the lateral cortex.

Patients 2 and 3 presented with defects measuring approximately 10 mm at the nonunion site.

### Nonunion characteristics

Two patients had aseptic atrophic nonunion, while one had a history of septic nonunion.

The presence of prior nonvascularized bone grafting varied: one patient had no prior grafting, one had undergone one procedure, and one had two prior procedures.

The number of previous surgeries ranged from two to three per patient.

**Table 1:** Preoperative patient data

Patients	Age	Sex	Site of nonunion	Nonvascul-arized bone grafts	Number of previous surgerie-s	Duration of nonu-nion	Presence of infection	Length of nonunion defect
Patient 1	34	male	right femur	0	3	30 months	yes	10 mm
Patient 2	43	male	left distal femur	1	2	42 months	no	10 mm
Patient 3	33	male	Right midshaft femur	2	3	108 months	no	10 mm

We included three patients with recalcitrant nonunion of the distal half of the femur. The inclusion criteria were patients with recalcitrant nonunion, such as septic nonunion or atrophic nonunion, with failed previous nonvascularized grafts.

### Preoperative assessment

To ensure that the pedicled medial femoral condyle (MFC) corticoperiosteal flap could reach the nonunion site without excessive tension, we calculated the transposition ratio for each patient preoperatively.

The transposition ratio was determined using the following formula:

$$Transposition{\text{ }}ratio\, = \,DMEB\,/\,DMELT$$

DMEB = Distance between the apex of the medial femoral epicondyle and the nonunion site (transferred bone).

**Fig. 1:**
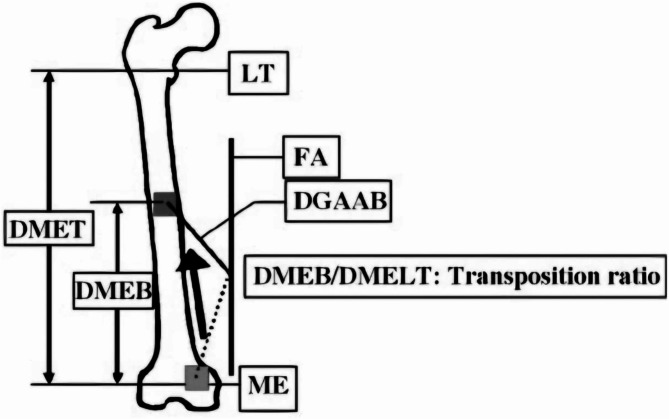
Anatomical landmarks used for measuring transposition ratio, DMEB and DMELT

DMELT = Distance between the apex of the medial femoral epicondyle and the apex of the lesser trochanter.

(Fig. 1 illustrates the anatomical landmarks used for these measurements.)

Patient-Specific Measurements are shown in Table 2.

All patients included had a transposition ratio ≤ 0.5, which was considered acceptable for pedicled transfer without undue tension on the vascular pedicle.

**Table 2:** Values of DMELT, DMEB, and transposition ratio

Patients	DMELT	DMEB	Transposition ratio
Patient no 1	34	13	0.38
Patient no 2	37	6	0.16
Patient no 3	30	11	0.37

Yoshida et al. [18] reported that a transposition ratio of less than 0.6 is suitable for pedicled medial femoral condyle corticoperiosteal flaps (Fig. 1). In this study, we included only patients with a transposition ratio of less than 0.5 to ensure that the medial femoral condyle corticoperiosteal flap would reliably reach the center of the nonunion site without tension.

### Preoperative evaluation and surgical planning

All patients underwent routine preoperative laboratory investigations, including complete blood count (CBC), coagulation profile, kidney function tests, and liver function tests, to ensure fitness for surgery.

### Surgical technique

**Fig. 2:**
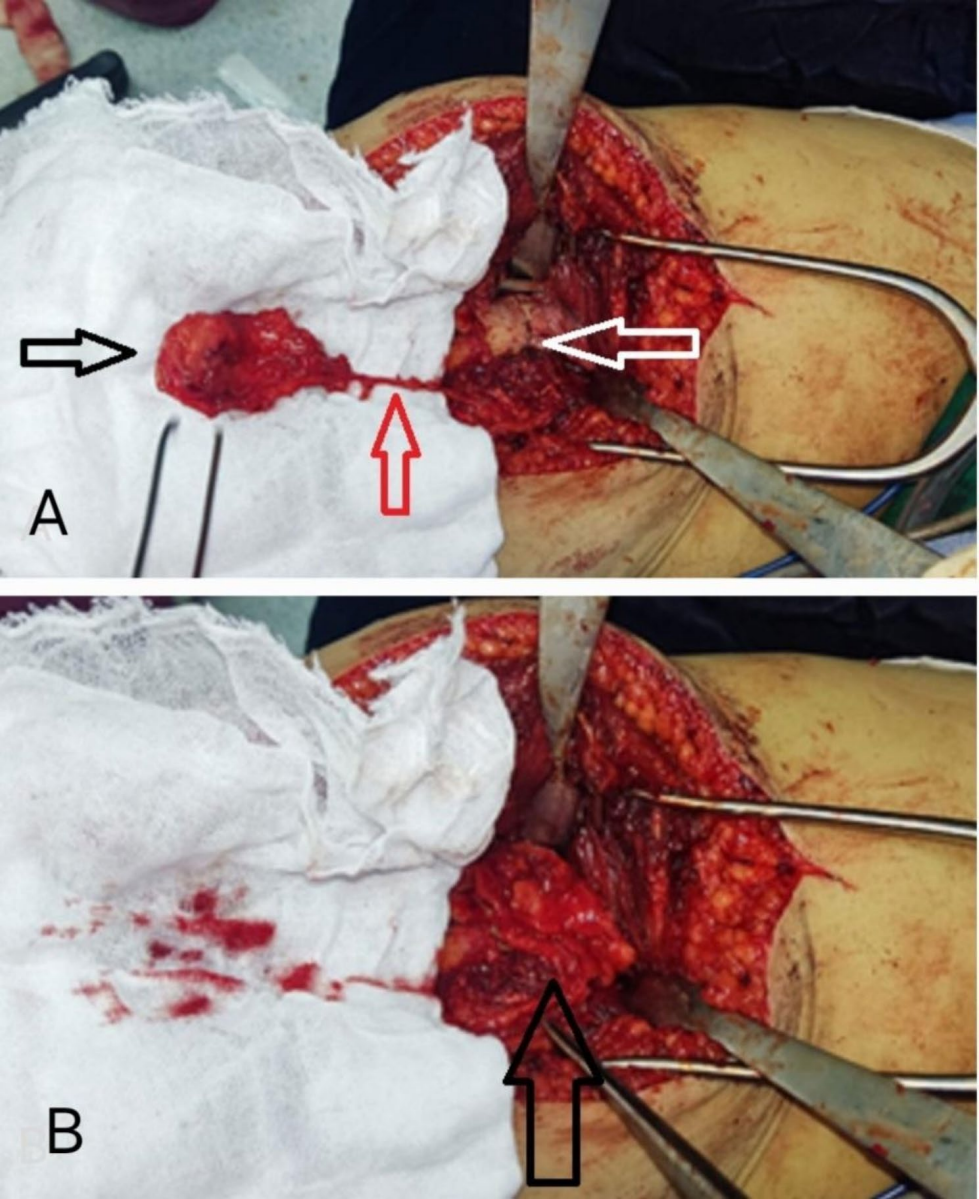
**A**: Medial femoral condyle flap after harvesting.Black arrow: medial femoral condyle corticoperiosteal flap. Red arrow: the flap pedicle. White arrow: nonunion site. **B**: Medial femoral condyle corticoperiosteal flap after being pedicled to the site of nonunion. Black arrow: the medial femoral condyle corticoperiosteal flap was wrapped around the nonunion site

In the patient with septic nonunion (Patient 1), a staged surgical approach was used: First stage involved removal of the previous implants and debridement of infected tissue, followed by placement of an antibiotic-loaded cement spacer to control infection. Second stage was performed after confirming infection control; the cement spacer was removed, rigid fixation was achieved using an interlocking femoral nail, an iliac crest bone graft was applied to fill the defect, and a pedicled medial femoral condyle corticoperiosteal flap was transferred to the nonunion site. In the other two patients (Patients 2 and 3), a single-stage procedure was performed, which included removal of old implants, resection of nonviable bony ends until punctate cortical bleeding (“paprika sign”) was observed, rigid fixation with appropriate implants, application of an iliac crest bone graft to fill the gap, and harvest and transfer of the pedicled medial femoral condyle corticoperiosteal flap to augment biological healing. The surgeries were performed by the first, third, and fourth authors at Cairo University Hospitals. Skin landmarks, including the medial epicondyle, vastus medialis, and adductor muscle, were drawn over the skin. The skin incision started from the medial femoral epicondyle, overlying the medial intermuscular septum between the vastus medialis anteriorly and the adductor group posteriorly, and ended at the area overlying the adductor hiatus. After incising the skin, the deep fascia was incised in line with the skin incision, and the vastus medialis was elevated from distal to proximal up to the adductor hiatus. Muscular branches to the vastus medialis were cauterized. The descending genicular vessels were identified over the medial intermuscular septum, lateral to the adductor magnus tendon, and were dissected up to the adductor hiatus. A small cortical shell attached to the overlying periosteum was harvested, in addition to the adjacent corticoperiosteal extension overlying the femoral metaphysis (Fig. 2).

The tourniquet was deflated to assess the bleeding and viability of the flap and to achieve adequate hemostasis. The flap was then rotated to cover the nonunion site and sutured to the surrounding muscles and bony ends using size 2 Vicryl sutures to avoid dislodgement. Closure was performed in layers with the placement of a suction drain. A high above-the-knee slab was applied postoperatively to prevent muscle contraction and potential dislodgement of the flap. Adequate rigid fixation was ensured in all cases to minimize strain at the fracture site, following biomechanical principles essential for bone healing. The combination of mechanical stability, iliac crest bone grafting for gap filling, and the vascularized corticoperiosteal flap aimed to enhance both the biological and mechanical environments for successful union. The patients were followed up with monthly X-rays and CT scans every three months. Union was confirmed by the appearance of bridging callus between the two bony ends on X-ray and was further confirmed by CT scan.

## Results

We included three patients in this study. The cause of nonunion was septic nonunion in one patient, while aseptic atrophic nonunion was the cause in two patients. The final fixation method was an interlocking femoral nail in one patient, a distal femur locked plate in another patient, and combined fixation in the third patient. Bony union occurred in all patients (union rate 100%), with an average time to union of 6.7 months. No mechanical failure was detected in any patient. Regarding complications, saphenous nerve injury and seroma occurred in one patient, while an incisional hernia at the iliac crest graft donor site was observed in another patient, as shown in Table 3.

**Table 3:** Postoperative patient data

Patients	Staged	Cement spacer	Final fixation	Time to union	Mechanical failure	Final fixation status	Relation to union	Follow up period	Complications
Patient 1	staged	yes	Nail femur	6 months	no	adequate rigid fixation	United	12 months	Saphenous nerve injury, seroma
Patient 2	Same session	no	Locked distal femur, small locked plate medially	6 months	no	Adequate rigid fixation	united	12 months	none
Patient 3	Same session	**no**	Nail femur and distal femur locked plate	8 months	no	adequate rigid fixation	united	12 months	Incisional hernia at the iliac crest donor site

The Lysholm Knee Score was used to assess the quality of life of the patients. We chose this score to evaluate knee function, as the flap was harvested from the medial femoral condyle and all three patients had fractures of the distal half of the femur. The average Lysholm Knee Score was 92 out of 100, ranging from 91 to 93, at the one-year follow-up.

### Case presentation

#### Patient 1

**Fig. 3:**
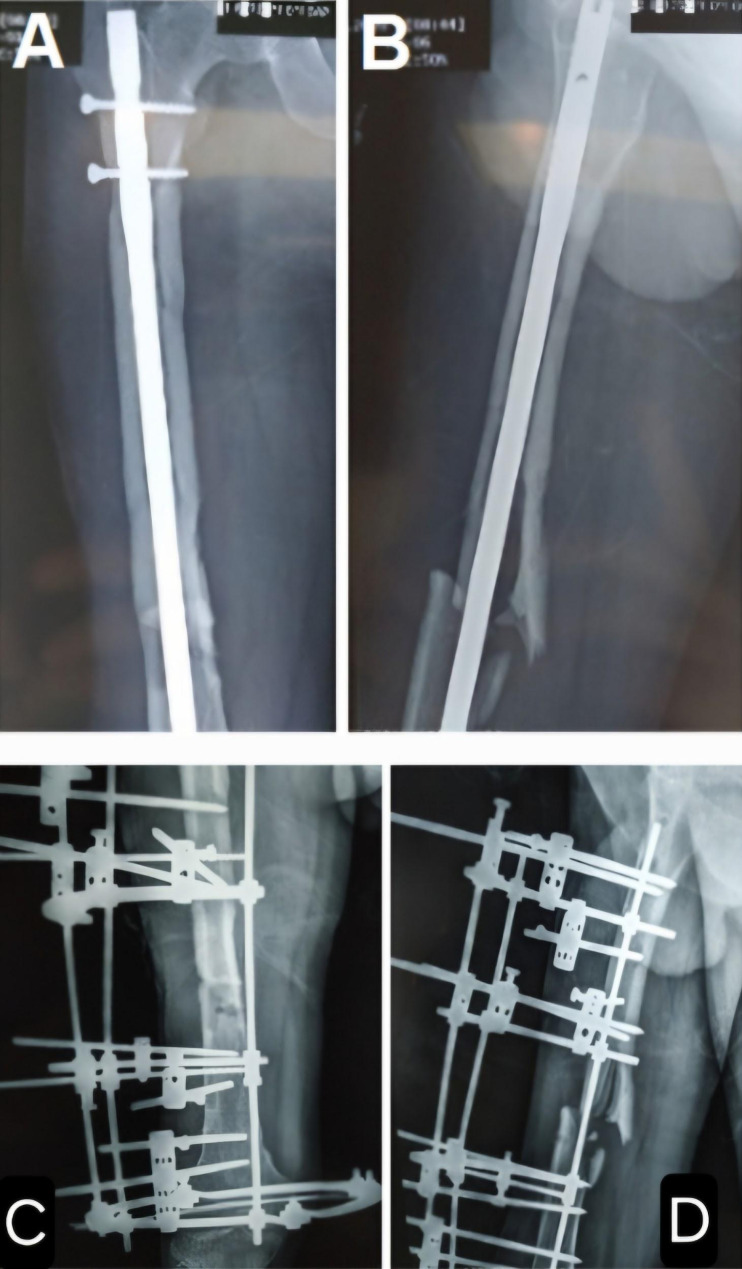
Infected nonunion of right nail femur, anteroposterior (**A**) and lateral (**B**) views.Ilizarov fixation of the right femur after nail removal, anteroposterior (**C**) and lateral (**D**) views

A 35-year-old male patient presented with infected nonunion of the left femur after interlocking nailing. The duration of the infected nonunion was 30 months. Initial management included debridement and Ilizarov fixation; however, the infected nonunion persisted (Fig. 3).

**Fig. 4:**
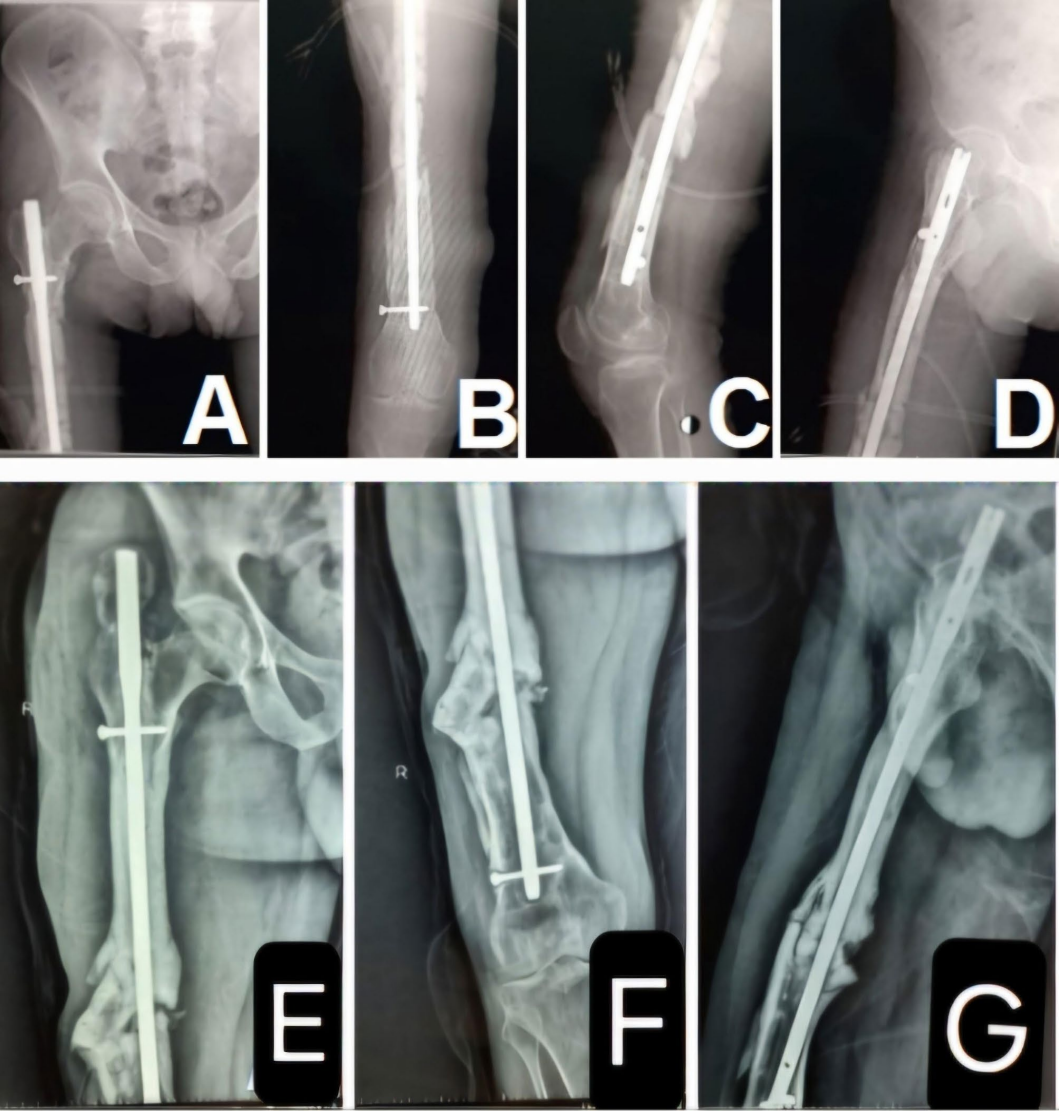
Cemented intramedullary nail femur from **A** to **D**. From E to G One-year follow-up of the cemented nail femur showing reactive bone formation at the nail entry site. **E**: Protrusion of the nail at the entry site. **F**: Lateral displacement of the cement spacer at the fracture site with bone contact at the medial cortex and a gap at the lateral cortex, which was filled by an iliac crest graft. **G**: The proximal locking screw was outside the hole

Subsequently, the Ilizarov fixator was removed, and sequestrectomy was performed, followed by insertion of a cemented intramedullary femoral nail and placement of a cement spacer at the fracture site. The patient did not follow up with us and returned after one year with the radiographs shown in (Fig. 4).

**Fig. 5:**
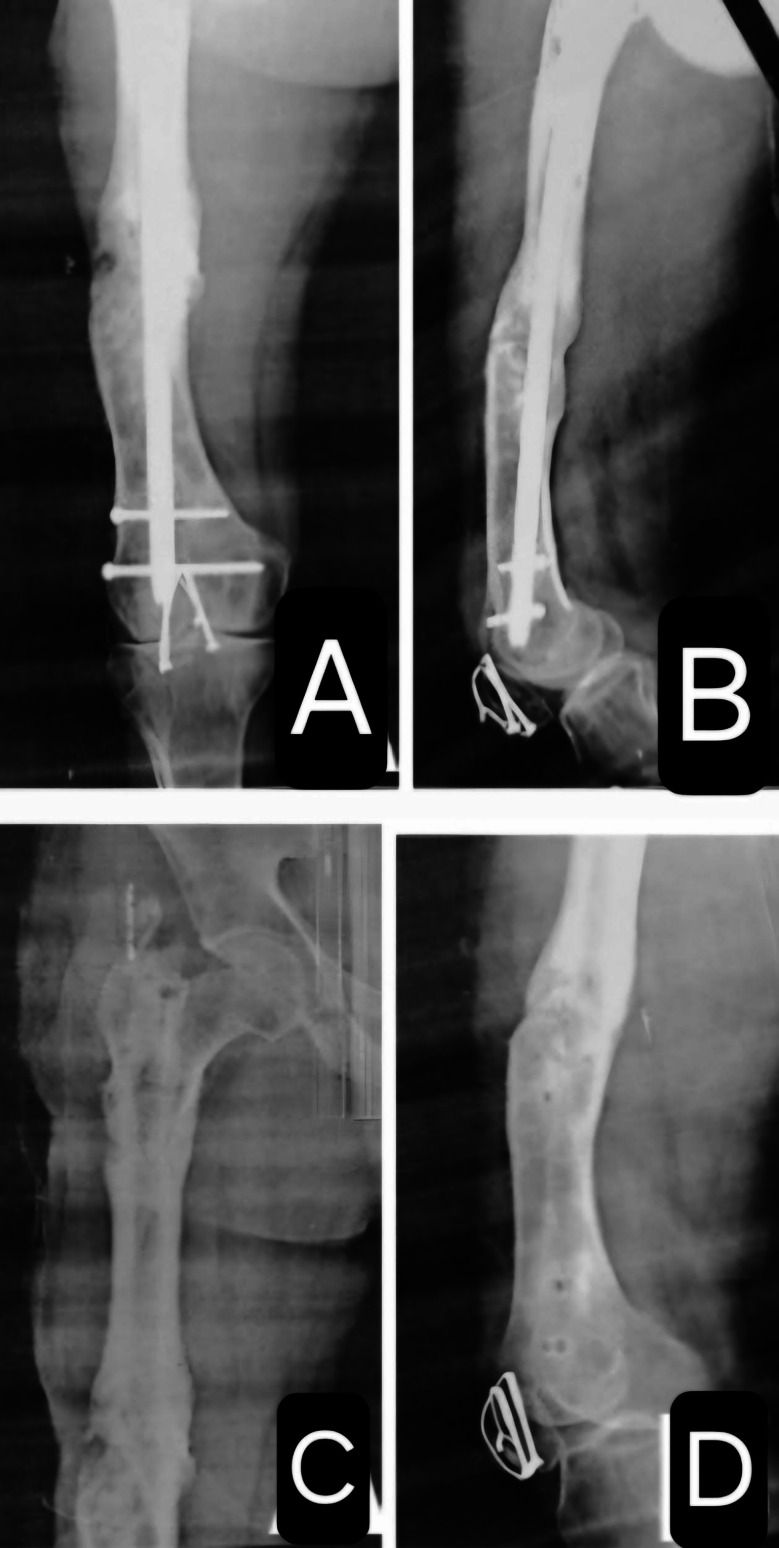
18 month follow-up image showing complete union, anteroposterior (**A**) and lateral (**B**) views. Complete fracture union after nail removal (**C** and **D**)

Operative debridement was performed again, along with removal of the cemented nail and cement spacer. The patient received intravenous broad-spectrum antibiotics for one week. Re-fixation was then performed using an intramedullary femoral nail, and a pedicled medial femoral condyle corticoperiosteal flap was applied. The patient was followed up monthly with X-rays and every three months with CT scans of the whole femur. Bony union was achieved after six months. However, the interlocking femoral nail was later removed due to recurrent infection, which presented as a discharging sinus (Fig. 5).

#### Patient no. 2

A 43-year-old male with a history of bilateral lower limb poliomyelitis presented with a recalcitrant atrophic nonunion of the distal femoral metaphysis, 42 months post-injury. The patient reported persistent pain and implant breakage. Imaging revealed a 10 mm bony gap at the fracture site.

**Fig. 6:**
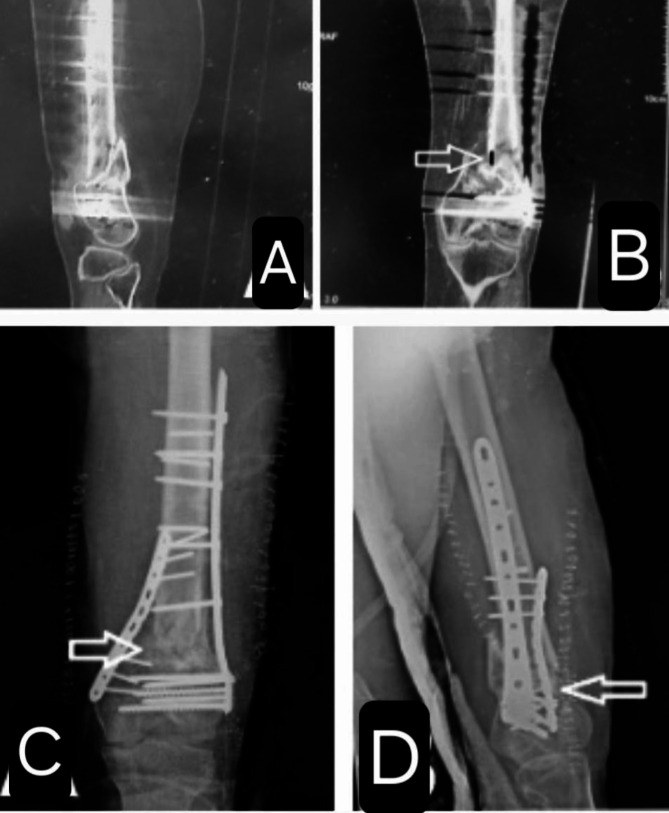
Preoperative and postoperative radiographs showing fracture nonunion and subsequent surgical intervention. Images **A** and **B** are preoperative views, with **A** showing the lateral view and **B** the anteroposterior (AP) view; in **B**, the white arrow indicates the nonunion gap, and the black line demonstrates a length of approximately 10 mm, as measured by the radiographic scale. Images **C** and **D** are postoperative X-rays, with **C** showing the AP view and **D** the lateral view; in both views, the white arrow indicates the medial femoral condyle corticoperiosteal flap applied to the anteromedial aspect of the femur

The previous implants were removed, and refixation was performed using a distal femoral locking plate complemented by an additional medial plate. The bone defect was filled with an autologous iliac crest bone graft. Additionally, a vascularized medial femoral condyle corticoperiosteal flap was harvested and applied to the anteromedial aspect of the femur. (Fig. 6).

**Fig. 7:**
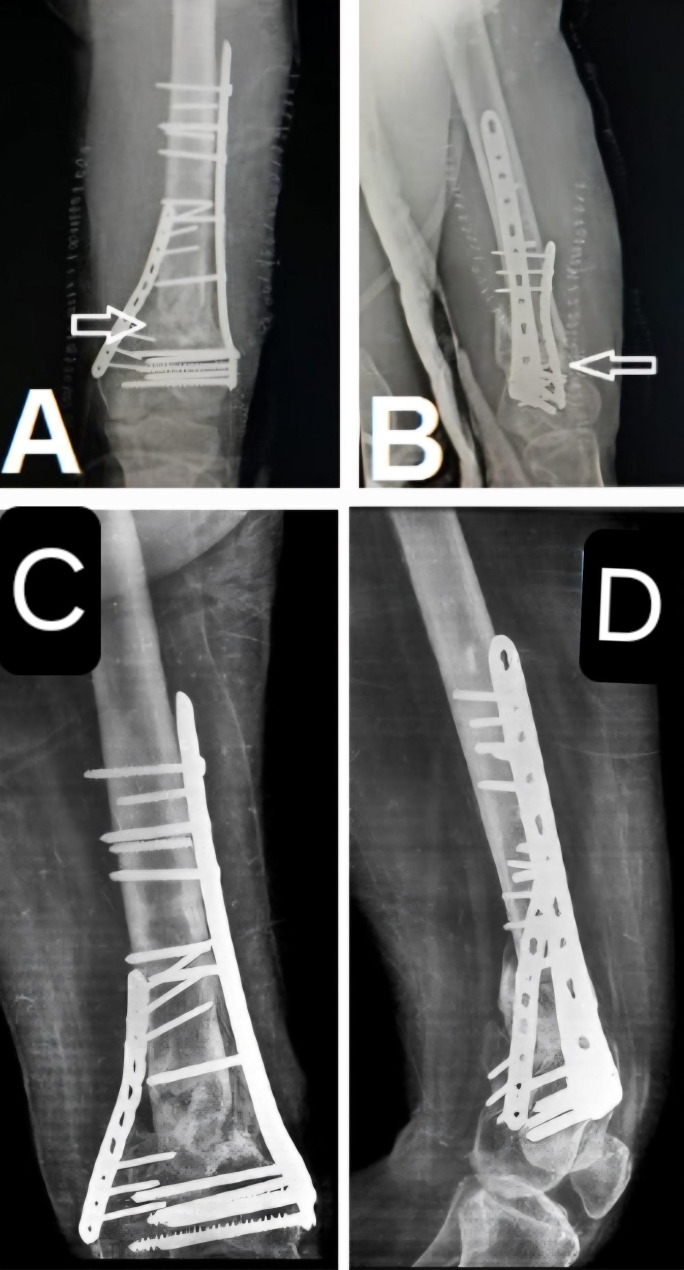
Postoperative X-rays showing the medial femoral condyle corticoperiosteal flap, indicated by the white arrows, in both the anteroposterior (**A**) and lateral (**B**) views. At the 6-month follow-up, the X-rays demonstrate fracture union in both views

Postoperatively, the patient underwent monthly follow-up with X-rays and CT scans every six months. Fracture union was achieved at six months. (Fig. 7).

**Fig. 8:**
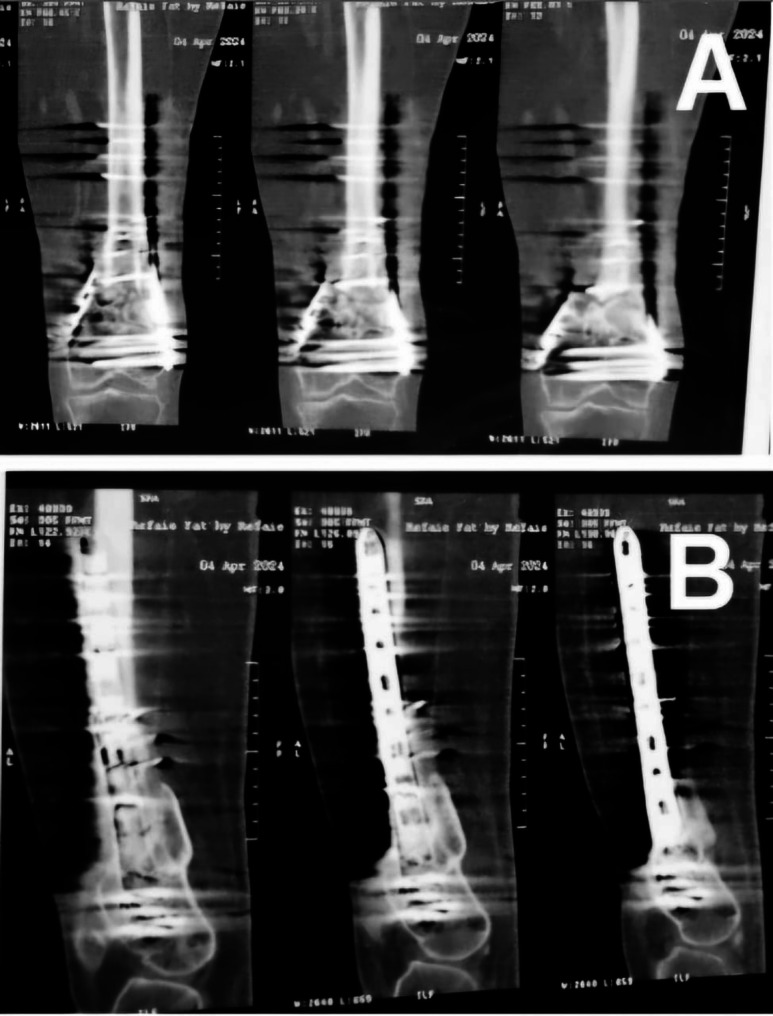
CT scan of the distal femur showing fracture union in both the coronal (**A**) and sagittal (**B**) views

At the one-year follow-up, the fracture site remained united with satisfactory clinical and radiological outcomes. (Fig. 8).

## Discussion

Management of recalcitrant femoral nonunion, particularly in the distal half of the femur, remains a significant clinical challenge due to the compromised biological environment, prior failed interventions, and the complexity of surgical reconstruction. In this context, the pedicled medial femoral condyle (MFC) corticoperiosteal flap has emerged as an effective treatment option, particularly for cases resistant to conventional methods.

In this study, we utilized the pedicled MFC corticoperiosteal flap for three patients with recalcitrant nonunion of the distal half of the femur. We achieved a 100% union rate, with an average time to bone union of 6.7 months. No mechanical failure was detected in any patient throughout the follow-up period. Our findings align with previous reports demonstrating the effectiveness of this flap in treating distal femoral nonunion. The technique provides the nonunion site with both vascularity and osteogenic cells, addressing two critical factors in bone healing. In cases of resistant nonunion, especially those with atrophic and devascularized bone ends, vascularized bone flaps such as the pedicled MFC corticoperiosteal flap are advantageous due to their capacity to re-establish vascularity and deliver osteogenic stem cells and growth factors to the fracture site. This biological enhancement, combined with adequate bony alignment and rigid fixation, facilitates early and reliable bone healing. Without rigid fixation, however, the risk of persistent nonunion remains high.

Our experience suggests that the pedicled MFC corticoperiosteal flap may be suitable for nonunions in the distal half of the femur. Recalcitrant nonunion of the proximal half of the femur is not amenable to treatment with this flap due to the limited arc of rotation and the vascular pedicle length. In our series, we observed that the flap reliably reached the midshaft of the femur but could not be transposed beyond this point. Therefore, it is essential to calculate the transposition ratio preoperatively for every patient, as recommended by Yoshida et al. [18], to ensure that the flap can reach the nonunion site. The medial femoral condyle corticoperiosteal flap is supplied primarily by the descending genicular vessels, which typically originate from the superficial femoral vessels at the level of the adductor hiatus. However, anatomical variations in this vascular supply exist. Silva et al. [14], in their cadaveric study of 30 specimens, found the descending genicular artery (DGA) present in 93.3% of cases, originating from the superficial femoral artery, while in the remaining 6.7%, the periosteum of the medial femoral condyle was nourished by the superior medial genicular artery (SMGA).

In our study, we confirmed the descending genicular artery as the predominant blood supply in all three patients. During flap harvesting, we included a thin cortical chip with the periosteum to preserve the cambium layer, in line with the technique described by Vegas et al. [19]. Their comparison of periosteal and corticoperiosteal flaps concluded that both methods are effective in promoting union in recalcitrant nonunion with small bone defects, though the corticoperiosteal flap may offer enhanced structural support and osteogenic potential.

Several studies support the efficacy of the pedicled MFC corticoperiosteal flap for femoral nonunions. Yoshida et al. [18] reported two cases of recalcitrant femoral nonunion successfully treated with this flap, achieving union in both cases. Similarly, in our series, all three patients achieved union using this technique, further supporting its effectiveness. Guzzini et al. [20] described a case of recalcitrant femur nonunion treated with removal of the original intramedullary nail, debridement of fibrous tissue at the nonunion site, re-nailing, and application of a pedicled MFC corticoperiosteal flap. Union was achieved at 3.5 months, with radiographic evidence of bridging cortices and the patient achieving full weight-bearing without pain.

Choudry et al. [21] reported a case series of 11 patients, three of whom were treated with pedicled MFC corticoperiosteal flaps for recalcitrant distal femur nonunion. Two patients achieved primary union, and one achieved secondary union after hardware exchange. Hamada et al. [22] also described three cases of femoral nonunion treated with the pedicled MFC corticoperiosteal flap, with all patients achieving complete union at 2, 1, and 1.5 months, respectively. They advocated for the use of this flap in distal femur nonunions without implant failure.

Ozdemir et al. [23] treated 13 patients with recalcitrant nonunion of the distal two-thirds of the femur using the pedicled MFC corticoperiosteal flap. The mean time to union was 5 months. Complications were minimal, including medial collateral ligament injury in one patient, hematoma in another, and seroma formation in two patients. They concluded that the pedicled MFC flap is an excellent option for recalcitrant femoral nonunions where larger vascularized flaps are not required. Its ease of harvest, lack of need for microvascular anastomosis, and minimal donor site morbidity make it an attractive surgical option.

Dekeyser et al. [2] reported on three patients with recalcitrant distal femur nonunion treated with the pedicled MFC corticoperiosteal flap, all of whom achieved union within 6 months. They recommended this flap as a reliable option with minimal donor site morbidity. Dhingra et al. [24] included five patients with recalcitrant distal femur nonunion in their study and supported the use of pedicled corticoperiosteal flaps due to their technical ease and reduced operating time. However, they highlighted challenges such as the inability to use a tourniquet and the necessity for meticulous dissection to avoid injury to the pedicle in scarred tissues. The absence of a tourniquet al.lowed better intraoperative identification of pulsating vessels with Doppler assistance.

Donor site morbidity is generally low with the pedicled MFC corticoperiosteal flap. Complications such as seroma formation and transient saphenous nerve affection were reported but typically resolved spontaneously. Major complications, including femur fractures and medial collateral ligament injuries, are rare. Scampa et al. [25], in their systematic review, confirmed these findings. However, Klarendic and Dovsac [26] reported an iatrogenic distal femur fracture following flap harvest in a 90-year-old female patient. In our study, we mitigated this risk by avoiding cancellous graft harvesting from the medial femoral condyle and instead used iliac crest cancellous grafts to fill any remaining gaps.

Our study adds to the growing evidence supporting the use of the pedicled medial femoral condyle (MFC) corticoperiosteal flap for recalcitrant distal femur nonunions. It emphasizes the importance of restoring vascularity in biologically compromised nonunion sites and demonstrates the feasibility of this technique without microvascular anastomosis. This offers an alternative for patients unsuitable for free vascularized fibular grafts or larger reconstructions. Additionally, our findings highlight the consistent vascular anatomy, practical surgical considerations such as flap reach, and the procedure’s low donor site morbidity. Importantly, it broadens the applicability of this technique to orthopedic surgeons without microsurgical expertise.

Despite the encouraging results of this study, several limitations must be acknowledged. First, the small sample size (three patients) limits the ability to generalize these findings to a broader patient population. Second, while all patients achieved union within a reasonable timeframe, the follow-up period was limited to one year, which may not capture late complications, including hardware failure or re-fracture. Third, we did not include standardized functional outcome measures to objectively assess improvements in pain, mobility, and quality of life. Fourth, the learning curve associated with flap harvesting and handling anatomical variations in the vascular supply may limit the widespread adoption of this technique. Finally, although donor site morbidity was minimal in our series, the potential for rare but serious complications, such as iatrogenic fractures, underscores the need for careful surgical technique and patient selection.

## Conclusion

In this study, the use of the pedicled medial femoral condyle corticoperiosteal flap for the treatment of recalcitrant distal femoral nonunion demonstrated promising outcomes, with all three patients achieving successful bone union within an average of 6.7 months and no mechanical failures observed. These preliminary results suggest that this technique may be a valuable option for addressing complex nonunions of the distal femur, particularly in cases where previous interventions have failed.

However, given the small sample size, the findings should be interpreted with caution. Further research involving larger, controlled studies is necessary to confirm the efficacy, safety, and long-term outcomes of this surgical approach. Additionally, careful patient selection and meticulous surgical technique remain crucial to optimize results and minimize complications.

## Data Availability

The research data is available with the corresponding author and will be available if needed.

## Abbreviations

MFC: Medial Femoral Condyle
CP: Corticoperiosteal
MFC-CP flap: Medial Femoral Condyle Corticoperiosteal Flap
DMEB: Distance from the Medial Epicondyle to the Nonunion Site
DMELT: Distance from the Medial Epicondyle to the Apex of the Lesser Trochanter
CT: Computed Tomography
CBC: Complete Blood Count
REC: Research Ethics Committee
AP: Anteroposterior
DGA: Descending Genicular Artery
SMGA: Superior Medial Genicular Artery
SFA: Superficial Femoral Artery
LT: Lesser Trochanter
ME: Medial Epicondyle
FA: Femoral Artery
MCL: Medial Collateral Ligament
Ilizarov: Ilizarov External Fixation Device
LKS: Lysholm Knee Score
ICBG: Iliac Crest Bone Graft
